# On Rattlesnake Poison, Rabies Canina, Etc.

**Published:** 1875-03

**Authors:** Alban S. Paine

**Affiliations:** Virginia


VOL. V.—MARCH, 1875.—NO. 3.

### Original Communications.

### ON RATTLESNAKE POISON, RABIES CANINA, ETC.

BY ALBAN S. PAINE, M.D., OF VIRGINIA.

From frequent observations, I have become satisfied that alcoholic drinks have been too much relied upon by the public for the cure of the bites of poisonous reptiles. I have, therefore, been induced to prepare this paper, with a view of presenting to the consideration of the medical profession a far more efficient remedy for the cure of these poisonous bites than either whisky, brandy or rum have proved themselves to be.

Before proceeding to the subject, however, I ask the privilege of submitting a few general and prefatory remarks. It is an undeniable fact that, if the science of chemistry is worth anything towards the elucidation of the complex and intricate science of medicine, it should teach us the chemical constituents, as well as the physiological and therapeutical action of our remedy upon the human system. Again, no one will dispute the importance of ascertaining the physiology of the pathological state before venturing upon a prescription for the cure of the morbid condition. Then, first, let us see whether the disease is functional or organic. If organic, whether it arises from plus or minus action of the organ or organs of the system found to be in a pathological condition. If only from functional derangement, does this functional derangement proceed from an excess of action, or from torpidity of the organs; or is it due to an excess of alkalescence, or acidity of the primæ viæ? Satisfied upon all these important points, we should next carefully consider and watch the "tendency to death" in our patient's case. Let us select and so chemically combine our remedies as to meet all the indications which may arise during the progress of the disease, and at the same time let them possess the power of obviating the ascertained "tendency to death." Thus, should we meet a case of rheumatism, in which it is obvious the "tendency to death" is from flagging of the circulation and vital powers, let us pathologically consider whether this disturbance to the general system, and consequent prostration, is due to torpidity, or to an excess of action in the secretory organs; or does it arise from an alkalescent or from an acid state of the primæ viæ. Finding it to be merely from functional derangement, rather than from any organic disease, and due to an excess of ascidity in the primæ viæ, and the "tendency to death" from sinking of the vital forces, or the heart failing to perform its functions—if we meet these two indications, we shall cure the rheumatism, viz.: counteract or neutralize the excess of acidity by the administration of an alkali or antacid, and meet the "tendency to death" by a stimulant. A prescription based upon such principles is not only rational, but may be said likewise to be scientific. Prescriptions upon other grounds are simply empirical. By an alkali we render the acid secretions inert and harmless to the general system; at the same time the alkali also possesses stimulating properties; will assist and sustain nature in her efforts to throw off disease. Now, when we consult our Materia Medica, we will find in that class of remedies denominated "stimulating alkalies" one calculated to meet both of the indications required to cure our rheumatic case, without subjecting us to the trouble of combining any other remedy along with it. I have reference to the "phosphate of ammonia."

If the case arises from an excess of alkali, exhibit an acid, and combat "tendency to death" by the appropriate remedies. This equally holds good, let the disease arise from whatever cause it may. Again, some persons seem entirely to overlook a well-ascertained fact that in some constitutions the "vis vitæ" is so strong that, if not interfered with in an incredibly short space of time, it will throw off disease without any remedial assistance. Indeed, I may go farther, and say that some constitutions are so tenacious of life, that I care not how carelessly and improperly they are prescribed for (if not killed downright by the most potent drugs), they will worry out most diseases and actually get well, so wonderful are the recuperative powers of their system.

Thus it sometimes happens that unreflecting and visionary persons, of otherwise fair intellects, prescribing for such constitutions (for it matters not what they take, they will get well), and mistaking the recuperative energies of such a system for the wonderful effect of some remedy they may have prescribed, commit their remedy to paper and hand it down to the public as one highly serviceable in curing this and that disease. Hence, we find our medical journals teeming with specifics for nearly all the ailments to which the human frame is liable, without any reference or respect being paid to chemical laws, or to pathological knowledge. I believe I could, by reasoning *a priori*, establish the fact that those cases of snake-bites reported cured by whisky, brandy and rum, have been rather prejudicial than beneficial to the interest of the public; occasioning the loss of many valuable lives that might have been saved by other remedies. The public becoming too well satisfied with, and relying too much upon whisky, a remedy which I am prepared to show is at best hazardous, and only capable of independently meeting a single indication, and that by averting the "tendency to death," possessing no curable or antidotal powers whatever. This has been done to the exclusion of another class of remedies far safer, more efficacious, and equally in the power of all grades of society to possess and to keep at all times in their houses, a remedy capable of meeting all and every indication of cure for these bites. I allude to "carb. of ammonia," or hartshorn. A residence of some thirty years in one of the most mountainous regions of Virginia, where the rattlesnake (species *crotalus horndus*), the copperhead and the moccasin are quite plentiful. I have had a fair opportunity of witnessing the effects of the poison when introduced into the system of the mammalia by the serpent's tooth. Besides, for six years I kept two large cages full of rattlesnakes, both male and female, in my office, and during three long years made many experiments with the poison upon the dog, cat, hen, etc., with a view to ascertaining the best and most prompt remedy in curing these poisonous bites. The more experiments I performed, the more satisfied I became of the value of hartshorn over all other remedies. If a remedy seemed to act well, I analyzed it, and I was sure to find it containing ammonia; its virtues depending on the amount of ammonia contained. The plantain, chickweed, actea, racemosa, etc., are all plentiful in ammonia, and were found valuable and superior to the much-lauded "bilious antidote."

The rattlesnake of the Blue Ridge mountains in Virginia is usually found from four to six feet in length, and from three to five inches in diameter. I have carefully dissected several, both male and female, from the infant snake with only a button, to the aged serpent with twelve rattles and a button. Dr. Rivers, in his interesting essay, truthfully says: "The symphises of the lower jaw is not united by bone, but by a ligament capable of great distention. It has a row of small, sharp teeth on each side of the jaws, which are very wide at the base, and without bony union. This arrangement enables it to hold on with one side of the lower jaw whilst the other side is advanced forward to take a fresh hold." "The throat is capable of almost indefinite distention, and whilst engaged in this operation a thick lubricating saliva pours out abundantly." "This, then, with the power of suction, enables it to perform such astonishing feats of deglutition." "One word with regard to the trail of this serpent. It is straight, whilst that of other serpents is zigzag." "He moves with a perpendicular motion—other serpents with a horizontal. So his trail may be distinguished from other serpents when crossing a road."

I know a road lying near the eastern base of the Blue Ridge mountains, in Fauquier county, Virginia, where, in the drouths of Summer, you can always find many trails crossing this road, in search of water. Here I can judge the size of any snake before he is captured. "The fangs, two in number, are situated in the upper jaw, one on each side, and when not in use, lay flat against the roof of the mouth, covered by a thin curtain like membrane." "These fangs are not fixed in bony sockets, but moved by strong muscles, and cannot go beyond the perpendicular; as they are erected, the base presses upon the sack of poison, which is injected through a hollow groove, extending from the base to near the point, which is exceedingly sharp." "These fangs are covered, and are much the form and shape of the talons of a bird of prey. At the root of the large fangs, and loosely attached to the gums, are found several rudimentary fangs, ready to take the place of the old ones, and commence to grow, should these latter by any means become broken off," or drawn out, which is easily done when this snake strikes a hard substance. When made to strike a soft piece of wood, he is apt to leave one or both fangs in it. As all the mammipers, she is viviparous and also prolific, bearing eighteen to twenty young at a birth. There is no doubt, until capable of caring for themselves, and in case of danger, her young find a hiding-place in the stomach of the maternal parent, and naturalists suppose the young feed upon the half-digested food found in the mother's stomach. I have seen, however, other snakes hide their young in this way.

The male is smaller, darker, more brilliant in color, and more symmetrically proportioned. The female is broader, larger, duller in color, more rusty, and more clumsily made than the male.—Whilst most observers describe the poison as a green, viscid substance, it always looked to me more like the honey of the wild honey bee than anything else I could compare it to. Nor could I ever find the quantity mentioned by others, two good, full drops being as much as I ever succeeded in obtaining from one snake. It is true, these snakes were kept in my office for five or six years, and often were captured for years before being killed for experiments, and as they never ate anything or indulged in copulation during this long period of confinement, this may have had something to do with the paucity of their poison. Yet, they looked as fresh and plump when killed as they did when captured in their mountain home. I put frogs, rabbits, etc., repeatedly in their cages, but never missed anything given them to eat in a single instance that I can remember. It is a singular fact, if you touch a rattlesnake with a twig of mountain ash, he becomes frozen, as it were, *instanter*. Dr. Rivers says: "A young man of my acquaintance, whilst examining the head of a snake, had the poison squirted on his hands, which, having sores upon them, gave him much uneasiness. But he sucked off the poison immediately, and escaped without any unpleasant effect. His tongue and teeth were *stained black* by it." My friend Dr. Dowler (than whom I recognize no higher authority) says: "Organic toxicological poisons are normal, and do not reproduce themselves in the organism, in which they induce toxicosis, as the poison of the crotalus, scorpion, hornet, etc. Pathogenic poisons, derived from an organic source, are abnormal, and tend to reproduce themselves, as the poison of syphilis, glandus, hydrophobia, etc. Toxicological poisons act with increased energy and danger on the young, the debilitated, the infirm, etc., females being more easily affected than males. The pathogenic poison of yellow fever, cholera, etc., as manifested in the same apartment, often destroys the powerful and robust, while the tender infant and the invalid are lightly affected. The treatment of toxicological poisoning is strictly antidotal, and with reference to throwing off, counteracting, rendering inert, and neutralizing the poisonous agent. The treatment of pathogenic poisoning, as in yellow fever, cholera, etc., is strictly antipathic, and is addressed to the morbid conditions present, and not to any assignable cause of those conditions."

Dr. Meade, a celebrated English physician, who devoted much time and study to the poisons of reptiles, was of the opinion that the cobra of India and the rattlesnake of America were more poisonous than any other of the poisonous reptiles. "Fortunately for those who live in districts infested with these serpents, they are not aggressive in disposition, and will seldom strike unless provoked or touched." This is true during the months of July and August, and arises more, I think, from a want of sight in the serpent than from any amiability of disposition on his part. When once aroused, however, and it does not take much to do that, he is the very personification of intense fury; throwing himself instantly in his coil, with eye flashing, tongue vibrating, head flattened and trembling, and rattles ringing incessantly, he is ready for battle, nor can he be intimidated. Once began, it is a battle of life or death with the rattlesnake. "It is then that a timid man will move cautiously behind him. But don't think to attack him from behind with safety, for he can throw himself his full length, and somewhat beyond his length, in any direction." In the "American Encyclopedia" it is stated "that he cannot, when taken by the hand around the neck, coil himself around the arm or use any constricting power." I have seen two valuable lives sacrificed to this popular error. I agree with Dr. Rivers when he says—another mistake from the same source—viz.: That he sounds his rattles some time before striking. This would indeed be a great blessing, but it is not so. Touch him, and he strikes instantly, and *then* sounds his rattles. The fact is, he sounds his rattles when he is enraged, and as long as he is enraged. These rattles are a unique and singular appendage, formed of hollow, flattened scales of horn, looped on to each other (but not joined together) so that they may be taken apart without breaking. The serpent has one for each year of his life. I think, as the serpent grows old, the rattle becomes brittle and breaks off, for some very large snakes are killed with very few rattles." In my opinion they shed their rattles every few years, for I have seen several serpents with every indication of age, great age, with a button and a single rattle. Dr. Rivers very aptly says: "These rattles are set in motion by the vibration of the muscles of the tail, and so rapid is it that you cannot perceive the motion. The sound is very much like the shrill notes of the cicada on a hot Summer's day—not so loud and shrill, and more of a buzzing sound; and the peculiarity of it is, you cannot locate the sound. He seems to play upon a harp of a thousand strings, and the sound seems to be in every direction around you."

Naturalists differ as to the uses of these rattles; some supposing that they were intended to warn his enemies, others to attract his prey, and he no doubt uses them for both purposes. They ring them when they are enraged or disturbed; they ring them to attract their prey, and perhaps to call their young. For my own part, I am satisfied that the Creator intended the rattles solely for the benefit of the serpent, and that the protection to man entered not into the calculation.

I can imagine the rattle of the snake as charming, as bewitching, as fascinating to the lower animals, as the dulcet strains of a "Blind Tom" are to the cultivated ear of the human race. I can imagine its natural enemy, the black-snake (the *Coluber*), about to make a vigorous attack, soothed, calmed, so completely pacified by the sweet jingle of the rattles, that the intended attack is abandoned. I can see the squirrel, the rabbit and bird fascinated by the music until it becomes an easy prey to the wiley charmer. I can believe the young called into the matutinal meal, or from threatened danger by the merry jingle of the mother's rattles. But I cannot imagine that those rattles were given unto the serpent in the interest of man. When the poison is introduced into the system of the mammalia, there is first a sudden and profound shock to the nervo-sanguiferous system, followed by swelling and pain in the part bitten, vertigo, dimness of vision, irregular contractions of the muscles, nausea, vomiting, relaxation, depression of the vital powers, syncope, and death. If we go to our Materia Medica again, we shall find there a remedy, a powerful poison, yet an acid, that feebly reddens litmus that is depressing upon the heart's action; given in an overdose, and you have a sudden shock to the nervo-sanguiferous system, nausea, vomiting, relaxation, collapse and death. As we find the physiological action of this acid (prussic) and the poison of the snake in its action upon the animal system are identical, it is natural and legitimate to believe their chemical constituents are also similar. Nature has, we know, placed this subtle poison in the peach kernel, it were no more remarkable to find it within the sack beneath the serpent's fangs; and we know one to be an acid, to imagine the other is also. When we bring to support this hypothesis the fact of the great efficiency with which hartshorn relieves these poisonous bites, stings, etc., I can but think that we add an additional link in the chain of argument to support our presumptions. If our arguments are correct, how easily, then, to explain the why and wherefore of our remedy. We can then explain the remedy's efficacy in curing these poisonous bites on well-established chemical laws. For if the poison of the snake is an acid poison, exerting a depressing influence upon the heart's action, we are bound by the laws of chemistry to combat it by a stimulating alkali; and we shall, whilst we avert the "tendency to death" by a stimulant, be able also to neutralize (and render innocuous) an acid poison by an alkali or antacid; for it is known to all that an acid and an alkali render each other inert and harmless by forming what is called a neutral salt. Were we to set all the antidotal powers of the hartshorn out of the question, and throw it back simply upon its stimulating properties, I should still (from its rapidity of action) claim for it the front rank in remedial virtues for the relief of these bites. Whisky, brandy, etc., are remedies certainly possessing no antidotal powers, and simply acting as stimulants; counteract, although more slowly, the "tendency to death,"

by forcing and keeping the heart in motion until nature has time to summon her forces and throw the poison from her system. If nature should prove weak, she may succumb (as I can establish she often does) under the depressing influence of the deadly poison, and a valuable life be sacrificed, when the poison would have readily yielded to the exhibition of the hartshorn. My first experiment with the poison was to produce the poisinous effects of it by inoculation. This I tried repeatedly upon cats, dogs, rabbits, and upon domestic fowls. Becoming emboldened by failure upon the lower animals, I vaccinated myself with a small quantity of the poison. In every instance of inoculation there was no perceptible effect produced. I therefore infer that the poison requires to be injected with force into the system. I regret the "hypodermic method" was not used in Virginia at that date. In my second experiment, I gave a large dog two drops of the undiluted poison; in fifteen minutes it made him "*howl.*" This dog howled incessantly for six hours, and appeared intoxicated for ten or twelve hours longer. I then mixed the poison and hartshorn in equal proportions and gave it to several small dogs. I thought it mildly *purged* them. I then took about one-third of a drop of the poison, properly mixed with hartshorn, worked it up with the crumb of bread, divided it into twenty pills and took two pills. I felt a glow about my stomach for several hours, and the next day my bowels were moved *gently*. I thought at one time I had succeeded in forming a mutual salt, by mixing certain proportions of the poison with aqua ammonia; but now I am satisfied one of my friends called my attention from the experiment a moment and slipped a small piece of iodide potassium in my mixture of these two substances. I had progressed this far in my experiments, when I had hardly time to kill all my snakes, before Gen. Blenker's Garibaldi Guards were upon me, and the destruction of my books, papers, MSS., etc., commenced. I have entirely forgotten about the taste and the smell of this poison.

Were I disposed to protract the length of this paper beyond reasonable limits, I could cite many recorded and unrecorded cases which have occurred in my own practice, and in the practice of others, serving to establish this assertion beyond controversy. I have in my own practice seen six cases of snake-bites, and two from spiders, in which hartshorn offered speedy relief, after whisky and brandy had entirely failed to do so.

This, to my knowledge, has frequently occurred in the practice of other physicians. For the sake of brevity, I will only allude to two cases—one from the bite of a spider, the other from the rattlesnake—which I deem sufficient, of themselves, to elucidate this part of my subject.

Some years since, a highly intelligent and prominent citizen of Fauquier county—more recently of Richmond county—in the act of shaving, was bitten upon the wrist by a poisonous spider. Although he was plied by an experienced and intelligent friend with the strongest French brandy, in doses rapidly succeeding each other, to the amount of sixteen ounces, yet I found him, one hour after the accident, as it were "in articulo mortis"—speechless, pulseless, and bathed from head to foot in a cold, clammy perspiration. Sixty grains carb. of ammonia, administered to him instantaneously, restored force to his circulation; and as soon as he could speak, he remarked to me that before the ammonia had been exhibited, he was suffering the most *excruciating* pain in his lumbar region. During our conversation, and whilst revolving in my own mind how often I should repeat the hartshorn in his case, he felt some return of pain, when he begged for the ammonia to be repeated. Sixty grains more of the hartshorn, then given, restored him to complete convalescence, only complaining for a few days of extreme tenderness of the soles of his feet. This symptom quickly yielded to the application of the unguentum stramonii.

I need not here more than allude to the sudden and untimely end of my friend, the accomplished Dr. Wainwright, of New York, the ripe scholar and accomplished gentleman, who was universally considered as being one of the first anatomists of his day. He had just received as a present a live rattlesnake from a medical friend, residing in Alabama. Being naturally of an inquiring turn of mind, and fond of zoölogy, he was engaged in examining the snake at the Carlton House, in the center of New York city, surrounded by a host of admiring friends, and a bright galaxy of medical talent, when he was bitten upon the hand; and although they *fed* him on the best of whisky and brandy, yet his generous and noble heart ceased to beat forever, under the depressing influence of the deadly poison.

*Dose and Mode of Administration.*—Sixty to one hundred grains of carb. ammonia, to be given in one ounce of water or milk, to be repeated in all cases in fifteen minutes until reaction is fully established. If the aqua ammonia is used, give from two teaspoonfuls to a tablespoonful and a half in three times its quantity of water, to be repeated as before directed. To prevent swelling, apply to the bitten part the following liniment, to be well rubbed in three times a day: Spts. hartshorn, 1 part; olive oil, 1 part; laudanum, $\frac{1}{4}$ part. If whisky or brandy has been previously used, administer your first dose of hartshorn in cider vinegar, or acetic acid diluted, for it occasionally happens that a person bitten by an insect or reptile exerting only slight poisonous properties upon the system, or the friends in their haste and alarm may have administered a great deal more whisky or brandy than was required; and if all danger from the bite is actually removed, yet the life of the patient may be seriously endangered from congestion of the brain, or from subsequent hyperæmia of the stomach. I have encountered such cases, and found the patient to be as "drunk as a lord," or as furious as a maniac—so much so as actually to be dangerous both to his medical adviser and to his attendants. Now, if you can by any feasible means induce him to swallow thirty to sixty grains of ammonia in three or four tablespoonfuls of vinegar or dilute acetic acid, he becomes at once sobered, as it were by magic. An ounce of the spts. menderi answers the same purpose, and is a specific for drunkenness from any of the alcoholic stimulants. We have now found the poison of these reptiles, insects, etc., to be not only rapid in their effects, but also to be powerfully selocant or depressant in their influence upon the system of the mammalia at large. We have also discovered, belonging to the Materia Medica, a powerful acid poison (hydrocyanic), displaying the same phenomena, and exerting the identical effect, when exhibited even in medicinal doses to the mammalia. Is it not, then, a fair inferenee to assume that the chemical constituents of the snake poison and this acid poison are synonymous, and that both are equally depressing, relaxing *acid* poisons, when introduced into the circulation of the mammalia?

During the preparation of this paper, a friend, Dr. R. C. Ambler, sent me "Coxe's Dispensatory," in which, at page 690, I find the following interesting letter, bearing the date of 1824. The editor says: "Although the poison of the viper has been experimented with, and even swallowed with impunity, it has not, we believe, ever been tried with a view to any medicinal powers it may possess. The following communication respecting the poison of the rattlesnake is of much interest, and may possibly, as the author suggests, lead sooner or later to its employment in medicine:

"'FAUQUIER, VIRGINIA, 1824.

"'After a review of animal, vegetable, mineral and aerial poisons, relative and positive, in their immediate and remote influence on the three grand functions—animal, vital and natural; seeing that the horse and dog are said to improve on arsenic; that it fails to poison the falco-ossofragus; seeing that swine devour in safety rattlesnakes, regardless of their numerous bites; and that carbonic acid gas, deleterious in the lungs, is innocent—nay, salutary—in the stomach, I made myself, *et alia*, subjects of experiments with the poison of the rattlesnake (*crotalus horndus.*) My moral views of mere principles and things forbade me pushing these experiments on others, whose safety is my professional study, (not the wild play of philosophic fancy, so far as I extended them on myself.) This animal substance is the true Sampson of the Materia Medica, and I anticipate the time when rattlesnakes will be reared for medicinal purposes, as the poppy and palmachristi are now. Old scholastic dogmas fly before modern science as chaff before the wind. I well remember when there was as much ceremony in giving a dose of calomel as christening a child in a country church. The effects of this poison are wonderful, as ethereal delights of long continuance, (say for days), whereas the effects of opium, hyoscyamus and lactucarium soon fade away; it reddens the blood, and makes the faded cheek to glow with the rose of youthful health; it is a great correcter of morbid resin of bile; it drives away typhus (*Τῦφος*), and replaces the mind on her native throne, to admire the beauties of creation, and inspire the soul with physico-theology.

"'N. B.—I mixed by friction in a glass mortar and pestle, the bags, venom and all, taken from the two teeth of a large and vigorous rattlesnake, with some cheese, and then divided the mass into one hundred pills, of which I occasionally took, sometimes one, at other times two, three or four pills a day; a general dropsy succeeded the first state of heavenly sensations, which has not even at this day fully gone off, being even now—March, 1827—subject to swellings in the evening. The diseases of the lymphatic and the arterial systems are never benefited by the use of the rattlesnake poison, but the nervous and muscular systems are speedily roused into action; palsy is much benefited; old rheumatisms are removed or relieved; the passions of the mind are wonderfully excited; delirium in typhus fever, attended with mutterings (typhomania), is almost immediately removed, and a serene mind, expressive of pleasure, follows. Melancholy is quickly changed into gay anticipations. Old sores are uniformly injured; on one occasion the old cicatrix opened, and was difficult to heal afterwards. An idiot became improved in intellect.

"'JAMES WESTWORTH WALLACE.

"'To JNO. R. COXE, M.D., Professor of Materia Medica in the University of Pennsylvania.'"

In my more youthful days I knew James Westworth Wallace, M.D., well. A lineal descendent of the renowned Sir William Wallace, a graduate of the celebrated University of Edinburg, he stood in the front rank of the medical profession. He was very *eccentric*; and his singular temper and his strange, eccentric habits made him enemies amongst his neighbors, who would otherwise have been his friends; but no one doubted his great ability as a physician. He cured numbers of cases of disease that were considered and pronounced incurable by other physicians of the first eminence in those days. He used the muriate of gold, in pillulæ form, a great deal in chronic uterine diseases. I know this remedy has of late years fallen somewhat into disuse; but as a tonic and alterative, I consider it to-day the most beneficial of the methods. In my opinion, progressively, he was *half a century ahead* of his day. He was very successful in his treatment of cholera and epilepsy; and I doubt not his Sampsom of the Materia Medica was the remedial agent he used in these cases; I neither doubt his truth or sincerity in what he states to Professor Coxe. I have said he was eccentric. He would ride none but a chickasaw horse, and rode one for a long time as *spotted* as a leopard. He wore in Winter a light coat, nankeen pants and a straw hat; in Summer, a *thick* cloth suit of clothes. Having a neighbor, remarkable for his want of personal pulchritude, the doctor summons him in haste. "What do you want, Doctor?" says the gentleman on his arrival. "Just *stick* your face in that bung-hole; I have been trying to turn the d——n cider to vinegar, and I just know your countenance will have the desired effect." * * * *Rabies Canina.* Let us for a moment inquire into the physical signs, or the phenomena, marking the introduction of the saliva of the mad-dog into the circulation of the mammalia, and see if we can discover any similarity to exist between the poison of the snake and the saliva of the mad-dog; and first let us inquire into its rapidity of action upon the animal economy. Is it rapid in its effects? No; if bitten by a mad-dog you may go mad in ten days or after the long period of twenty-four years. Is it relaxant or depressant on the heart's action? No; the circulation, as a general thing, is about normal at intervals (during paroxysms), being actually increased in force and frequency. Is the "tendency to death" from relaxation or depression of the muscular fibres of the heart and general system? No; death is due to a tendency to muscular contraction, thus giving rise to convulsions or irregular clonic contractions of the muscular fibres, particularly those muscles surrounding the throat and parts of deglutition. These eccentric muscular contractions render the act of deglutition of solids painful, and of liquids next to impossible. As the disease progresses, the sight of liquids become sufficient to bring on these clonic spasms of sufficient force to convulse the whole frame, without any attempt at deglutition being made. Hence the name of hydrophobia, or dread of water. Reasoning, then, from analogy, we would not, then, be justified in believing their chemical constituents to be identical. Should we not rather be led to infer that they were the *very* opposite of one another, both as regards their chemical constituents and their electrical condition? If one is an acid poison, the other must be an alkaline one. If one is in a negative electrical condition, the other must be in a positive state. We are confident the poison of the rattlesnake is acid, feebly reddening litmus. We also believe saliva of the mad-dog to be alkalescent, and would give the alkaline reaction.

If our hypothesis is correct, the poison of the snake and the saliva of the dog mixed together would present a soponaceous appearance, entirely inoffensive in its character, and if swallowed by a human subject, might move the bowels as a mild aperient medicine. Having found, then, the very antipodes of one another as characterized by the symptoms displayed by each in their influence upon the circulation of the mammalia, as well as upon all other portions of the system, it must follow that they are opposite in their chemical combinations; and it follows, as a matter of course, the treatment must also be antagonistic. To meet the indications of cure for rabies, we are therefore forced to select an acid remedy possessing a relaxing or depressing influence upon the muscular fibres, rather than any stimulating properties. According to this view of the subject, the administration of acetic acid for the cure of rabies appears to be much more in accordance with scientific principles than the recommendation of either hartshorn, whisky or brandy. But before we decide finally upon this point, let us examine a little more minutely into the "tendency to death" from this poisonous saliva, as well as into our Materia Medica. If we can procure the rattlesnake poison, we shall give it the preference over all other remedies; but not having a supply on hand, and forced to select another, let us consult our Materia Medica; possibly we may discover there some remedy meeting all the indications of cure much more efficiently than either vinegar or acetic acid. Let us again inquire what are the prominent indications to be met and controlled. We have no need of a stimulant, for we find the circulation not at all depressed by this poison, but at times (during the paroxysms) actually increased in force and frequency; the muscles not relaxed, but evincing a proneness to take on irregular clonic contractions. If we will consult the Materia Medica, we shall find there, under the head of virulent poisons, a remedy possessing just such properties, viz.: An acid feebly reddening litmus, and exerting a powerful depressing influence upon the muscular contractions of the heart, as well as upon all the muscular fibres of the human frame—so much so that, if taken in too large a dose, the heart ceases to beat, just as we have seen to be the case from the depressing influence of the rattlesnake poison. This acid is termed hydrocyanic or prussic, and, according to my way of thinking, it is second only to rattlesnake poison to meet all the indications of cure for a mad-dog bite. I profess to no experience upon this branch of my subject, never having seen a mad-dog, or a person bitten by one, in my life. I have been led to the above conclusions upon this subject—first, from seeing the identity of effect upon the circulation produced by the prussic acid and the poison of the snake, etc.; second, by the opposite phenomena, as displayed by the saliva of rabies, as recorded by different authors. It is fortunate for the human family that rabies is comparatively a rare disease, not more than one dog in every one hundred being really mad that are supposed to be so. Every young dog secreting saliva freely, suffering from some indigestible substance in the stomach, or from the irritation incident to "teething," is pronounced mad. Every dog that has convulsions, arising from all the numerous causes that may produce them in dogs, as well as in the human subject, is doomed to "die the death of a mad-dog."

The causes of rabies are thought by some to be produced by excess in barometrical alternations of heat and cold. Others imagine the disease to be the sequence of excessive pain from toothache. But it is my earnest opinion that the cause is from the want of sexual intercourse. In some countries where they allow so many sluts to live for the accommodation of dogs (at a small fee), rabies is found to be unknown. Recorded experience teaches us the fact that not more than one person in every twenty-four who are actually bitten by a mad-dog ever show any symptoms of hydrophobia. This probably arises from one of two causes: either the fang of the dog is often cleansed of the saliva in passing through the clothes, or from the fact that animal poisons, like the emanations arising from vegetable decay, require a predisposition to be furnished by the human for their successful propagation. I incline, however, to the former opinion, as we find animals (cattle, etc.) much oftener evincing symptoms of hydrophobia, after a bite from a suspicious dog, than human beings, whose skin is protected to some extent by their clothing. This view of the subject seems partially to explain the reason why cures by the mad-stone and other "fallacious specifics" yet find advocates among the more ignorant and credulous populace. Those cases bitten by a dog not actually mad, and those if bitten by a dog actually mad, still would never evince any signs or symptoms of rabies, are those that are represented as being cured by the application of the mad-stone, "cutting the bitten piece out," and other equally ridiculous prescriptions. In my childhood I have heard of the wonders performed by the madstone: if the dog was not mad, the stone would drop off, and could not by any means be made to stick to the bite; but if the dog was really mad, then the stone would cling and draw until it would become filled with a green poison imbibed from the wound. I imagine this mad-stone to be a porous anhidrite of alumina, possessing great affinity for moisture—not a magnesian stone, as Professor Nathan R. Smith, M.D., supposes. I dug up one on my farm a few years ago, beautifully striped with yellow, black and white stripes; drop it into a glass of water, and you could hear it singing and humming like a large bug in the adjoining room, so rapidly did it attract water.

Some may urge, as an objection to the prussic acid in these cases, its well-known poisonous properties; but how can we reasonably expect successfully to contend against such a disease without the most potent weapons? Again, every physician is presumed to be aware of the fact that in acute pain a person can take an amount of opium or other drugs, with perfect immunity from danger, that would speedily prove fatal were he in sound health and free from pain. Also, of the fact that where the human system is depressed by the influence of these poisonous snake-bites, what enormous doses of whisky, brandy or other stimulants it will bear without any perceptible effect being produced therefrom. This seems to be a firm and fixed law existing between disease and its antidote. But fortunately, we are not called upon, in treating hydrophobia, for any sudden and high dosing, either to meet the indications of cure, or to arrest the "tendency to death," for the indications rather demand a saturation of the system by small and repeated doses of our remedy, aiming thereby gradually to lessen the force of the circulation and the tendency to muscular contraction, than by suddenly raising or relaxing them by a large dose of our antidote. Thus we have ample time afforded to exhibit small medicinal doses of this potent drug, carefully watching its effects, until the system is gradually brought under its influence. Were I prescribing for a case of hydrophobia, I should also use the acid, mixed with some oleaginous substance, topically to the bitten part.

I have thrown out these crude suggestions, hoping they may prove of some benefit to the profession, and may serve to call to this interesting field for investigation abler minds than mine.

